# S100A11 (calgizzarin) is released via NETosis in rheumatoid arthritis (RA) and stimulates IL-6 and TNF secretion by neutrophils

**DOI:** 10.1038/s41598-021-85561-3

**Published:** 2021-03-16

**Authors:** Adéla Navrátilová, Viktor Bečvář, Jiří Baloun, Dres Damgaard, Claus Henrik Nielsen, David Veigl, Karel Pavelka, Jiří Vencovský, Ladislav Šenolt, Lucie Andrés Cerezo

**Affiliations:** 1 Institute of Rheumatology, Na Slupi 4, 12850 Prague, Czech Republic; 2 Department of Rheumatology, 1st Faculty of Medicine, Charles University, Prague, Czech Republic; 3 First Orthopaedic Clinic, 1st Faculty of Medicine, Charles University, Prague, Czech Republic; 4 Institute for Inflammation Research, Center for Rheumatology and Spine Diseases, Copenhagen University Hospital, Rigshospitalet, Copenhagen, Denmark

**Keywords:** Rheumatoid arthritis, Autoimmunity, Cell death and immune response, Immune cell death, Rheumatoid arthritis, Chronic inflammation, Neutrophils, Cell death, Rheumatoid arthritis, Immunology

## Abstract

S100A11 (calgizzarin), a member of S100 family, is associated with several autoimmune diseases, including rheumatoid arthritis (RA). Neutrophil extracellular traps (NETs) are implicated in the pathogenesis of RA and in the externalization of some S100 family members. Therefore, we aimed to determine the association between S100A11 and NETs in RA. For this purpose, the levels of S100A11 and NETosis markers were detected in the RA synovial fluid by immunoassays. The expression of S100A11 by neutrophils in the RA synovial tissue was assessed. Neutrophils isolated from peripheral blood were exposed to S100A11 or stimulated to release NETs. The levels of NETosis- and inflammation-associated proteins were analysed by immunoassays. NETs were visualized by immunofluorescence. We showed that S100A11 was expressed by the neutrophils in the RA synovial tissue. Moreover, S100A11 in the RA synovial fluid correlated with several NETosis markers. In vitro, S100A11 was abundantly released by neutrophils undergoing NETosis compared to untreated cells (p < 0.001). Extracellular S100A11 increased the secretion of IL-6 (p < 0.05) and TNF (p < 0.05) by neutrophils but did not induce NETosis. This study demonstrates, for the first time, that the release of S100A11 is dependent on NETosis and that extracellular S100A11 augments the inflammatory response by inducing pro-inflammatory cytokines in neutrophils.

## Introduction

Rheumatoid arthritis (RA) is a chronic, immune-mediated, systemic disease characterized by persistent synovial inflammation that leads to irreversible structural damage and joint failure. Both genetic and environmental factors contribute to an increased risk for RA development. Among the various cell types involved in the pathogenesis of RA, neutrophils play an essential role in the onset and progression of RA^1^. Neutrophils are the most abundant leukocytes in the bloodstream and in the synovial fluid of RA patients^2^ and possess significant immunomodulatory, cytotoxic and antimicrobial functions^3^. Neutrophils are capable of forming neutrophil extracellular traps (NETs) as a host defence mechanism, and this process is termed NETosis^4,5^. The dysregulation of NETosis has been reported in a variety of autoimmune diseases^6–8^, including RA, in which NETs represent a major source of citrullinated proteins—autoantigens that are thought to drive the autoimmune processes underlying anti-CCP-positive RA^9^. Moreover, recent studies indicate that the components of NETs, such as nuclear material and granular and cytoplasmic proteins, not only serve as mechanism of pathogen clearance but also can act as damage-associated molecular patterns (DAMPs)^10,11^, thereby augmenting inflammation or inducing further NETosis in a positive-feedback loop^11,12^.

S100 protein family consists of small calcium-binding proteins with helix-loop-helix motifs ("EF-hand type") involved in the regulation of a variety of cellular functions and implicated in numerous pathological conditions incuding cancerogenesis^13^. Certain S100 proteins, upon being released from damaged or activated cells, act as DAMPs by virtue of their ability to drive inflammatory processes via receptors for advanced glycation end-products (RAGE) or Toll-like receptor 4 (TLR-4)^14–16^. In the past decades, extensive studies have linked S100 proteins to RA [reviewed in^17^]. Our group has recently reported a role of the less known S100 protein family member S100A11 (calgizzarin) in the pathogenesis of RA^16^. We found that S100A11 expression was upregulated in patients with RA and associated with disease activity, inflammation and autoantibodies against citrullinated proteins (anti-CCP)^16^. In addition, we observed enhanced S100A11 expression and secretion by the synovial fibroblasts and mononuclear cells of RA patients^16^. However, the contribution of neutrophils, as potential producers of S100A11, to RA has not previously been assessed. Gravius et al. identified S100A11 as one of the neutrophil-associated proteins within inflamed periprosthetic tissue^18^. Moreover, recent studies indicate that some S100 proteins can be released by neutrophils via NETosis^19,20^. Taken together, these findings prompted our current study to assess the association of S100A11 with neutrophils and NETosis in RA.

## Results

### S100A11 is synthesized and secreted by neutrophils from RA patients

**Figure 1:**
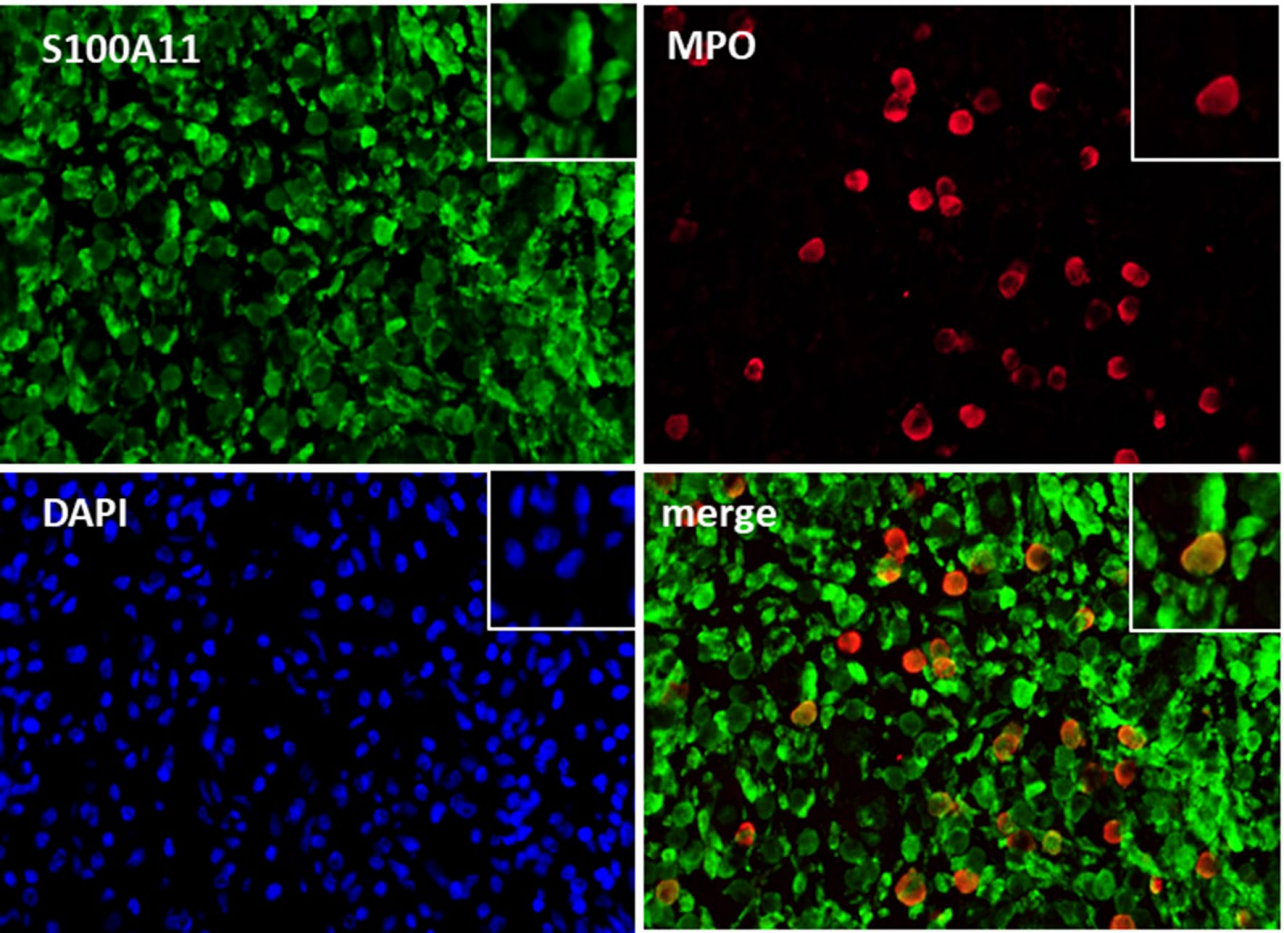
S100A11 is expressed by infiltrating neutrophils in synovial tissue from RA patients. Double immunofluorescence staining shows the co-localization of S100A11 (green) and myeloperoxidase (MPO) (red). Cells were visualized using an Olympus BX53 microscope with Olympus cellSens Standard imaging software V1.18. (https://www.olympus-lifescience.com/en/software/cellsens/). Representative images are shown at ×400 magnification, and cellular details are shown at 1000x (A) (n = 8).

Using immunofluorescence staining, we demonstrated that myeloperoxidase (MPO) -positive neutrophils infiltrating the RA synovial tissue expressed S100A11 (Fig. 1). In order to determine if S100A11 was released from neutrophils into the extracellular space we analysed the levels of S100A11 in the supernatants from neutrophils isolated from peripheral blood transefered into in vitro conditions. We found that the in vitro secretion of S100A11 by neutrophils from RA patients was comparable to that of neutrophils from healthy controls after a 4 h (0.09 (0.08–0.11) vs. 0.08 (0.03–0.23) ng/ml; p = NS) or 24 h (1.24 (0.90–1.67) vs. 0.86 (0.62–1.55) ng/ml; p = NS) incubation interval. These results indicate that there is a basal secretion of S100A11 by neutrophils in vitro.

### Synovial fluid S100A11 is associated with the anti-CCP positivity and with the markers of NETosis in RA patients

**Figure 2:**
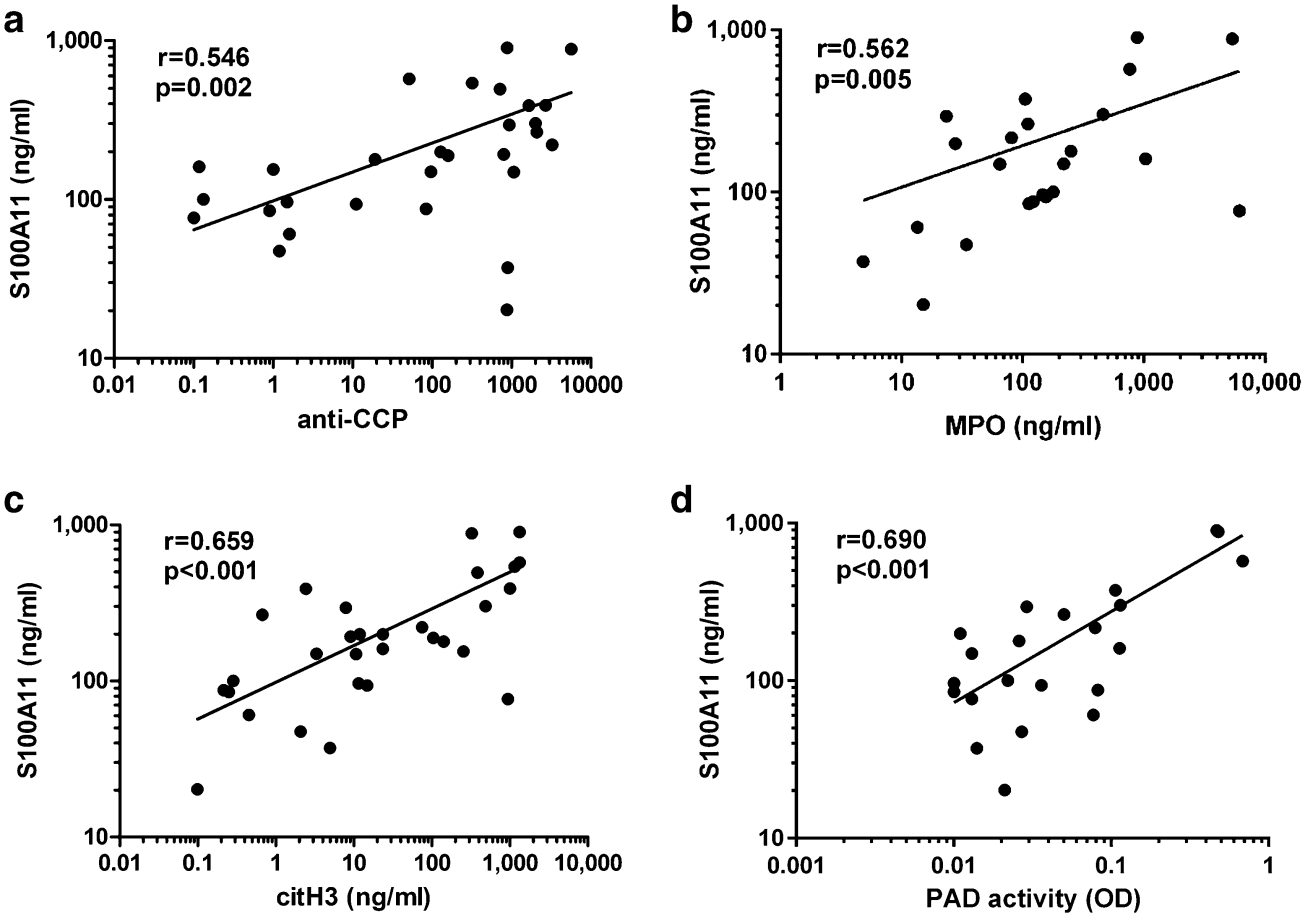
The synovial fluid levels of S100A11 significantly correlate with the levels of anti-CCP in the synovial fluid of RA patients (**a**). S100A11 is associated with the levels of myeloperoxidase (MPO) (**b**), citrullinated histone 3 (citH3) (**c**) and with the activity of peptidyl-arginine deiminase (PAD) enzymes (**d**) in the synovial fluid of patients with RA (n = 23–30).

Here we demonstrate that S100A11 significantly correlated with the levels of anti-CCP in the synovial fluid of RA patients (r = 0.546, p = 0.002) (Fig. 2a). Moreover, the levels of S100A11 were significantly upregulated in the anti-CCP positive compared to anti-CCP negative patients (265.70 (149.30–495.10) vs. 94.87 (72.63–156.00) U/ml; p = 0.003). Given the clear relation of S100A11 to citrullination, we further aimed to evaluate whether the externalization of S100A11 by neutrophils may be linked to the process of NETosis. Therefore, we analysed the levels of S100A11 in association with markers of NETosis, such as free MPO, citH3 and PAD enzyme activity. The levels of S100A11 in the synovial fluid from RA patients significantly correlated with the levels of MPO (r = 0.562, p = 0.005) and citH3 (r = 0.659, p < 0.001) (Fig. 2b,c). Moreover, the level of S100A11 was strongly associated with the activity of PAD enzymes in the synovial fluid (r = 0.690, p < 0.001) (Fig. 2d).

### S100A11 is externalized via NETosis

**Figure 3:**
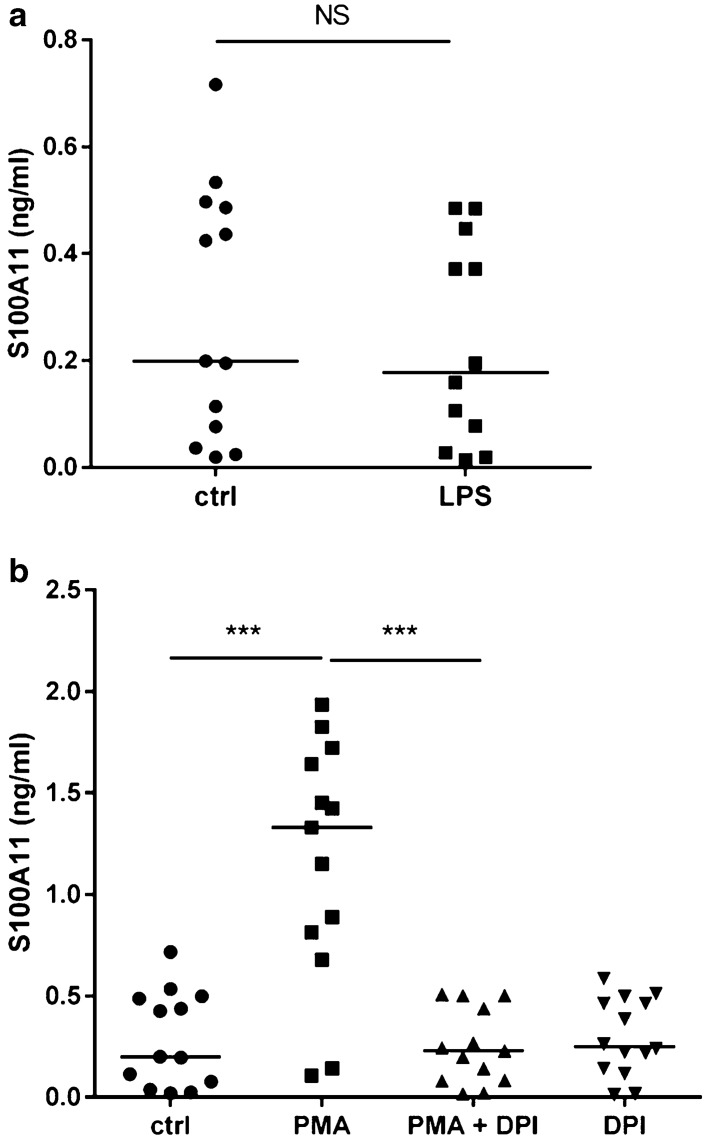
Neutrophils from RA patients exposed to lipopolysaccharide (LPS) did not exhibit increased S100A11 secretion compared to unstimulated neutrophils (4 h), (**a**). Stimulation of NETosis by phorbol 12-myristate 13-acetate (PMA) for 4 h upregulated the secretion of S100A11 by neutrophils. Pretreatment with diphenylene iodonium (DPI) before PMA eliminated the formation of NETs and thereby the release of S100A11 (**b**). *Ctrl* unstimulated control, *NS* non-significant. The horizontal line represents the median. ***p < 0.001.

**Figure 4:**
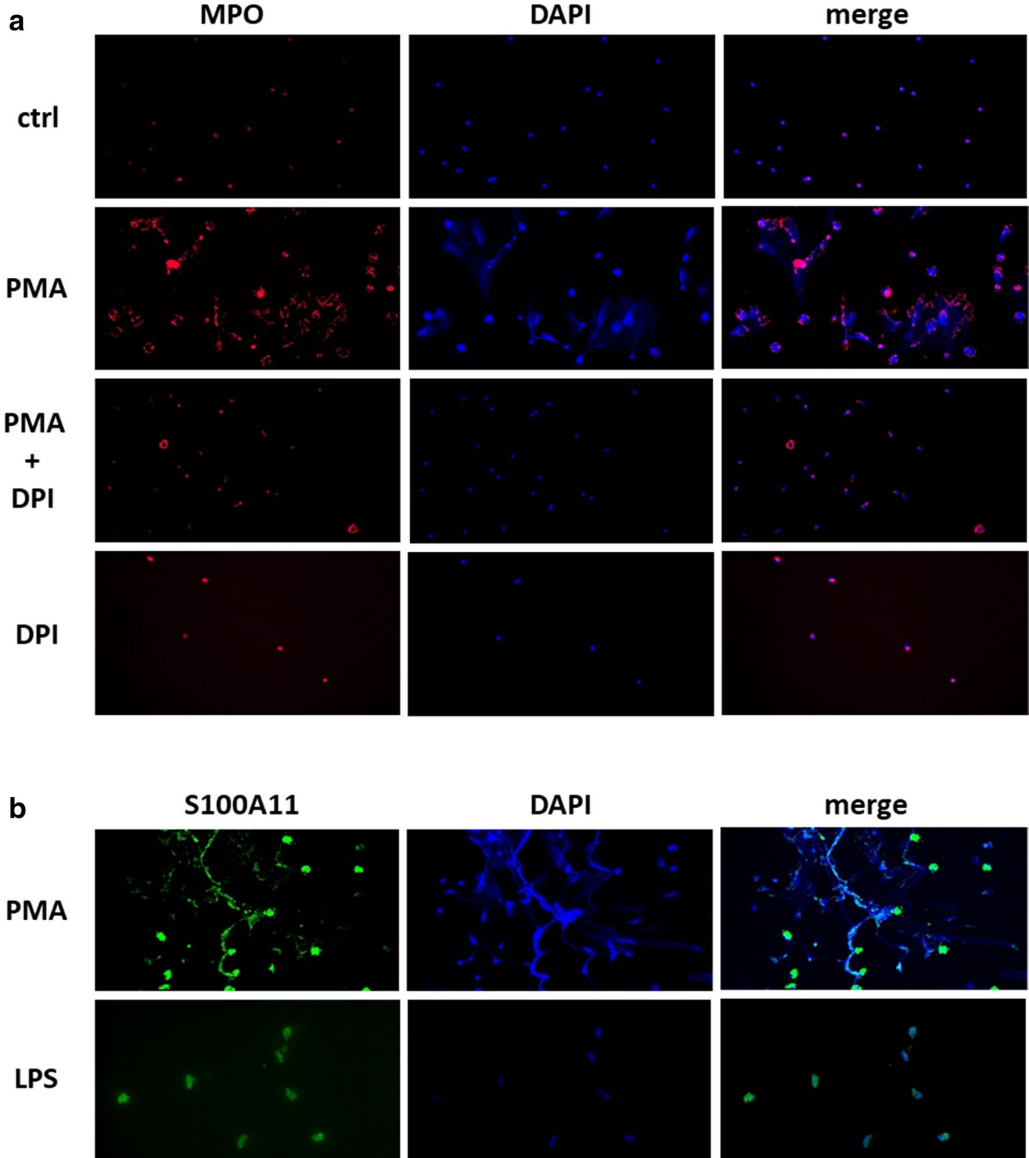
S100A11 was externalized during NETosis. Neutrophils stimulated for 4 h with phorbol 12-myristate 13-acetate (PMA), diphenylene iodonium (DPI) or a combination of both were stained for DNA (DAPI; blue) and myeloperoxidase (MPO; red) to visualize the induction of NETosis (**a**). Neutrophils stimulated with PMA exhibited NETosis (**a**,**b**) accompanied by S100A11 (green) release (**b**). In contrast, pro-inflammatory stimulation with lipopolysaccharide (LPS) did not lead to NETosis or S100A11 secretion by neutrophils (**b**). Ctrl, unstimulated control. Cells were visualized using an Olympus BX53 microscope with Olympus cellSens Standard imaging software V1.18. (https://www.olympus-lifescience.com/en/software/cellsens/). Representative images are shown at ×400 magnification (n = 7).

Given the association of S100A11 with NETosis, we aimed to examine the conditions under which neutrophils release S100A11. Neutrophils isolated from peripheral blood of RA patients were stimulated with phorbol 12-myristate 13-acetate (PMA) to trigger NETosis or with lipopolysaccharide (LPS) to induce an inflammatory response. First, neutrophils were treated for 4 h with LPS from *E. coli* serotype O26:B6, which, based on the literature^21^ and our results (Supplementary Fig. S1), induces an inflammatory response but not NETosis. As shown in Fig. 3a, LPS-treated neutrophils did not exhibit increased secretion of S100A11 compared to unstimulated neutrophils (0.18 (0.04–0.43) vs. 0.20 (0.04–0.48) ng/ml; p = NS). Second, stimulation of neutrophils with PMA induced a significant release of MPO (833.20 (708.19–1240.13) vs. 105.97 (73.33–125.61) ng/ml; p < 0.05) and S100A11 (1.33 (0.75–1.68) vs. 0.20 (0.04–0.48) ng/ml; p < 0.001) (Fig. 3b), indicating that ongoing NETosis was accompanied by S100A11 release. In this experiment, diphenylene iodonium (DPI, a selective inhibitor of NADPH oxidase) eliminated the externalization of NETs and thereby the release of S100A11 (Fig. 3b). NETosis induction and S100A11 release by neutrophils from RA patients were also visualized by immunofluorescence staining (n = 7) (Fig. 4a,b).

### Extracellular S100A11 induces an inflammatory response of neutrophils but not NETosis

**Figure 5:**
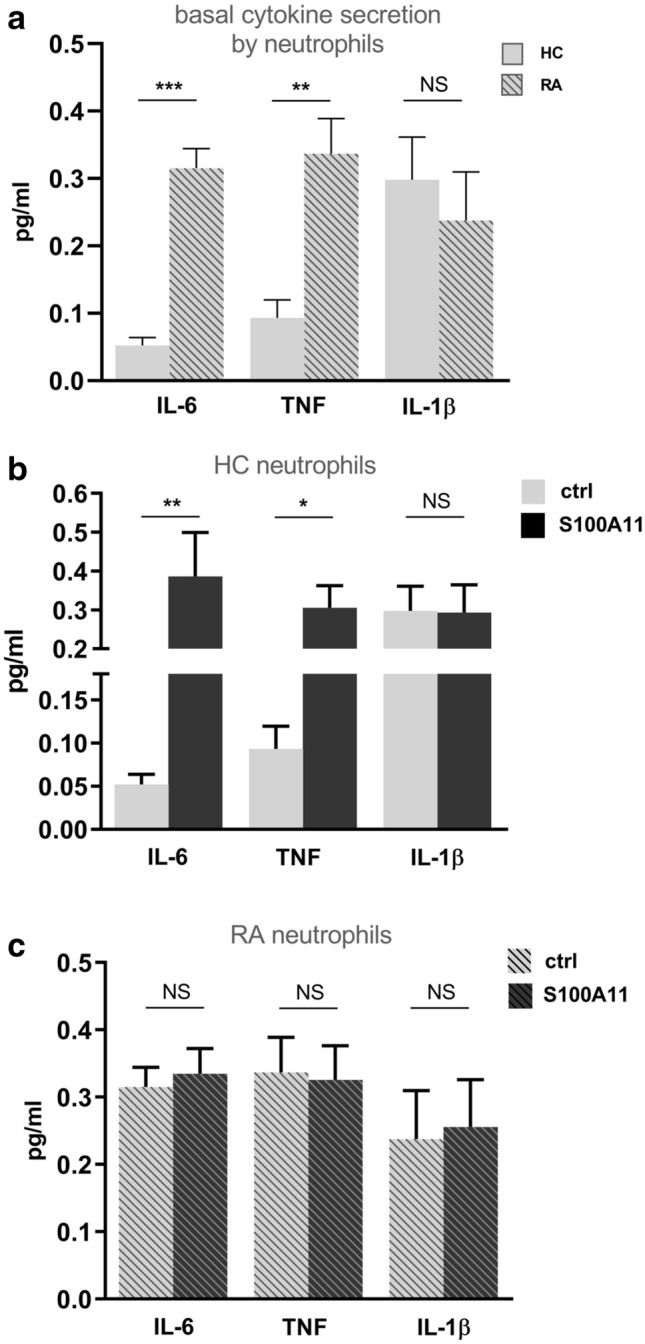
Neutrophils from RA patients showed increased basal secretion of IL-6 and TNF in contrast to neutrophils from healthy controls (HC) (**a**). S100A11-treated HC neutrophils (n = 8–10) exhibited significantly increased secretion of the pro-inflammatory cytokines IL-6 and TNF, but not IL-1β, compared to unstimulated neutrophils (**b**). In contrast, treatment with S100A11 did not affect the release of cytokines by neutrophils from RA patients (n = 6–7) (**c**). Neutrophils were stimulated for 4 h time intervals. *HC* healthy control, *ctrl* unstimulated control, *NS* non-significant. The data are represented as the mean ± SEM. *p < 0.05, **p < 0.01, ***p < 0.001.

First, we observed an increased basal secretion of pro-inflammatory cytokines interleukin (IL)-6 and tumour necrosis factor (TNF) in peripheral blood RA neutrophils in contrast to neutrophils from healthy controls (IL-6: 0.29 (0.26–0.35) vs. 0.04 (0.03–0.08), pg/ml; p < 0.001 and TNF: 0.33 (0.23–0.47) vs. 0.07 (0.03–0.14), pg/ml; p < 0.01) (Fig. 5a). The basal levels of IL-1β did not differ between RA and healthy neutrophils (0.19 (0.08–0.40 vs. 0.22 (0.19–0.48), pg/ml; p = NS). Consequently, neutrophils from healthy individuals exposed to recombinant S100A11 exhibited significantly increased secretion of the pro-inflammatory cytokines IL-6 (0.28 (0.12–0.59) vs. 0.04 (0.03–0.08) pg/ml; p < 0.05) and TNF (0.23 (0.19–0.42) vs. 0.07 (0.03–0.14) pg/ml; p < 0.05), but not IL-1β (0.17 (0.11–0.48) vs. 0.22 (0.19–0.48) pg/ml; p = NS) compared to unstimulated neutrophils (Fig. 5b). In contrast, neutrophils from peripheral blood of RA patients were not affected by S100A11 in terms of the secretion of IL-6 (0.31 (0.25–0.38) vs. 0.29 (0.26–0.35) pg/ml; p = NS), TNF (0.36 (0.14–0.42) vs. 0.33 (0.23–0.47) pg/ml; p = NS) or IL-1β (0.20 (0.07–0.44) vs. 0.19 (0.08–0.40) pg/ml; p = NS) (Fig. 5c). Also, the potential of S100A11 to induce NETosis has been tested in vitro by measuring the levels of MPO released by RA and healthy controls ‘ peripheral blood neutrophils exposed to S100A11. RA neutrophils exhibited no elevation of MPO levels after treatment with S100A11 when compared to untreated cells (115.80 (73.53–365.20) vs. 108.00 (86.55–167.40) ng/ml; p = NS). Similarly, no changes in MPO secretion were observed in S100A11 treated neutrophils from healthy controls compared to unstimulated cells (68.55 (22.81–98.26) vs. 61.29 (24.35–80.33), suggesting that S100A11 does not trigger NETosis.

## Discussion

Here, we show, for the first time, that S100A11 is externalized in complex with NETs and induces the secretion of the pro-inflammatory cytokines IL-6 and TNF by neutrophils from healthy individuals.

Over the past decade, a number of studies have focused on the role of S100A11 in tumorigenesis and metastasis^22–25^. Recently, our research group described an association of S100A11 with the pathogenesis of several rheumatic autoimmune diseases^16,26,27^. It is well established that neutrophils are the first cell type recruited to sites of inflammation^11^ and that neutrophils are an important source of S100 alarmins^28,29^. We showed that S100A11 is expressed by neutrophils infiltrating the synovial tissue of RA patients. This is consistent with other S100 proteins and with the data of Gravius et al., who identified S100A11 as a peptide associated with neutrophils^18^. Furthermore, according to our observations, S100A11 is spontaneously released by neutrophils in vitro. Although the in vitro secretion of S100A11 by the neutrophils from RA patients and healthy controls were comparable, it is likely that in the pathological environment of RA synovial fluid, neutrophils constitute an important source of S100A11. This hypothesis is supported by the fact that neutrophils are the most abundant white blood cells in the synovial fluid^30^, and the synovial fluid of RA patients contains high levels of S100A11^16^. Of note, the enhanced release of S100A11 by neutrophils from both healthy controls and RA patients over time is most likely caused by the short life span of neutrophils and their increased cell death.

As mentioned above, S100A11 accumulates in the synovial fluid of RA patients and is associated with the levels of anti-CCP^16^, indicating a link between S100A11 and the processes of citrullination and enhanced NETosis. This hypothesis is supported by the correlation of S100A11 with activity of PAD enzymes in RA synovial fluid. Indeed, in this study, the levels of S100A11 were related to the levels of MPO and citH3 in the synovial fluid, suggesting that all three components are released during NETosis in RA. In fact, several studies have shown that certain S100 proteins can be externalized via NETosis^19,20^. Consistently, we demonstrated that neutrophils from RA patients undergoing PMA-induced NETosis released abundant levels of S100A11.

To assess the ability of “non-NETotic” neutrophils to actively release S100A11, neutrophils were treated with LPS of serotype O26:B6, which is, based on our results and literature^21,31^, capable of triggering an inflammatory reaction without inducing NETosis. Consistent with peripheral blood mononuclear cells (PBMCs) and synovial fibroblasts^16^, and in contrast to chondrocytes^32^, neutrophils did not secrete S100A11 after stimulation with LPS, suggesting that the release of S100A11 is dependent on neutrophil death. We can also assume that the mechanism of S100A11 secretion and its triggers, similar to those of other S100 proteins^33^, are cell-type specific.

We have recently demonstrated that extracellular S100A11 acts as a DAMP recognized by PBMCs^16^. Since DAMPs can induce NETosis^11^, we hypothesized that S100A11 externalized by NETosis forms a positive feedback loop by stimulating other neutrophils to release NETs. Consistent with literature^34^, RA neutrophils spontaneously released higher levels of MPO compared to healthy cells, indicating enhanced NETosis in RA. However; no direct effect of S100A11 on NETosis was observed in neither RA nor healthy neutrophils. Provided that multiple cytokines can trigger NETosis^12^, its is not excluded that S100A11 may indirectly contribute to NETosis regulation via cytokine induction. Indeed, we have demonstrated that S100A11 significantly enhances the secretion of pro-inflammatory cytokines in neutrophils from healthy individuals but not in RA neutrophils. In addition to that, the basal cytokine secretion was markedly higher in RA neutrophils compared to healthy cells. It is well established that RA neutrophils are functionally different from healthy neutrophils, as demonstrated by their ehanced lifespan^35^, increased phagocytosis and superoxide anion production^36^ or enhanced NETosis and degranulation^36^. Moreover, recent study implies that healthy neutrophils challenged by proinflammatory conditions up-regulate cytokine production^37^. Taken together, we assume that the increased basal cytokine secretion in RA neutrophils is related to their activation status caused by the exposure to the pathological inflammatory millieu in RA. Consequently, the „altered phenotype “RA neutrophils may not be sensitive to additional pro-inflammatory stimuli such as S100A11. Finally, we can speculate that the pro-inflammatory effect of S100A11 on neutrophils may be significant only at the onset of RA before the chronic infammation develops. In addition to that, our results emphasize the function of S100A11 within the context of inflammation; however, it must be noted that S100A11 exerts less potent immunomodulatory functions than other S100 proteins^15,16^. As previously demonstrated by Dempsey et al., there is a difference in the molecular structure of S100A11 when compared to other S100, specifically in the arrangement of certain helices of the „EF- hand“ motif^38^. It is well established that S100 proteins lack enzymatic activity and their regulatory function depends on calcium binding which leads to conformational change creating binding site for target proteins. Thus, it can be speculated that the specific molecular structure of S100A11 may affect the binding of target proteins and thereby the immunomodulatory potential of S100A11. Moreover, in contrast to other S100 family members, S100A11 exhibits lower affinity to calcium ions^13^.

Least but not last, the posttranslational modifications may modulate the function of S100A11. Although not explored in this study, the activity of S100A11 released by NETs may depend on its phosphorylation state, as shown by Schenten et al. for S100A8/9^19^. Moreover, recent study on S100A3 protein revealed that citrullination of S100A3 can lead to conformational changes from dimeric to oligomeric forms^39^, which in some S100 proteins represent the biologicaly active froms^40^. Therefore, further studies are warranted to assess the form of S100A11 released during NETosis.

Some limitations of this study should be taken into consideration. First, a relatively small number of samples were included in our study. Second, we used blood neutrophils for the in vitro experiments, but synovial fluid neutrophils would have been more appropriate. Third, in addition to PMA, there are several other inducers of NETosis that were not included in our study. Fourth, the role of neutrophil degranulation in S100A11 externalization was not assessed. Further studies that evaluate the role of S100A11 in a joint environment are needed.

Despite these limitations, this study reveals, for the first time, a link between S100A11 and NETosis and demonstrates a pro-inflammatory effect of S100A11 on human neutrophils. Moreover, the presence of S100A11 in NETs may contribute to the immunogenic character of NETs and amplify the local inflammation in RA joints.

## Conclusion

Taken all together, our data indicate that the release of S100A11 by neutrophils is linked to NETosis. Moreover, S100A11 augments the inflammatory response by inducing IL-6 and TNF secretion in healthy neutrophils.

## Materials and methods

### Patients

The study included 30 patients with RA (12 females and 18 males, mean age ± SD 53.2 ± 13.4 years) who provided paired samples of synovial fluid and peripheral blood. The peripheral blood used to isolate neutrophils was collected from early RA patients from the Institute of Rheumatology (n = 12–14) or from healthy controls (n = 8–10). The patient and healthy control characteristics are shown in Table 1. Patients with early RA were either prior to (treatment naïve) or no longer than six months after the start of methotrexate or glucocorticoids (prednisone ≤ 10 mg per day). The patients with RA met the American College of Rheumatology criteria for the diagnosis of RA^41^. The study was approved by the Ethics Board of the Institute of Rheumatology. All methods were performed in accordance with the relevant guidelines and regulations and written informed consent was obtained from all participants in this study.

**Table 1:** Characteristics of patients with rheumatoid arthritis (RA) and healthy controls (HC).

Patients characteristics	Serum/synovial fluid	Peripheral blood neutrophils	
	RA	RA	HC
Number of patients	n = 30	n = 14	n = 10
Gender (males/females)	18 / 12	5/9	4/6
Age (years)	53.2 ± 13.4	60.9 ± 14.4	34.2 ± 13.5
Disease duration (years)	8.0 ± 9.5	7.7 ± 5.6	–
DAS28	4.36 ± 1.17	–	–
CRP (mg/l)	26.07 ± 31.67	5.6 ± 5.5	2.6 ± 0.5
Anti-CCP positivity (%)	18 (60%)	8 (57%)	–
RF positivity (%)	17 (57%)	7 (50%)	–
Drugs (csDMARDs/GCs)	28 / 20	8/7	–
Biological DMARDs	6*	0	–

*Anti-CCP* anti-cyclic citrullinated peptide antibody, *CRP* C-reactive protein, *DAS28* disease activity score in 28 joints from CRP, *csDMARDs* conventional synthetic disease-modifying antirheumatic drugs, *GCs* glucocorticoids, *RF* rheumatoid factor IgM.

*Out of 6 patients, 2 were treated with tocilizumab, 1 with ofatimumab, 1 with humira, 1 with Enbrel and 1 with anti-IL-17 therapy. Data are expressed as the mean ± SD unless stated otherwise.

### Laboratory analysis of serum and synovial fluid

To analyse the levels of S100A11 and its associaton to the markers of inflamamtion or NETosis, paired samples of peripheral blood and synovial fluid were collected from RA patients with clinically indicated knee arthrocentesis, and the samples were immediately processed and stored at -80 °C. Before analysis, the synovial fluid samples were treated with hyaluronidase (Hylase Dessau; Riemser Arzneimittel, Greifswald, Germany) for 30 min at 37 °C and centrifuged. Serum was separated from the peripheral blood from the RA patients and stored at − 80 °C until use. The clinical assessment of RA activity was performed in 28 joints using the disease activity score based on C-reactive protein (DAS28-CRP). The CRP levels were determined by turbidimetry using an Olympus Biochemical Analyser (Olympus, Tokyo, Japan), and the serum anti-CCP and IgM rheumatoid factor (IgM-RF) levels were determined using the corresponding enzyme-linked immunosorbent assay (ELISA) kits (TestLine Clinical Diagnostics, Brno, Czech Republic).

### Isolation of human peripheral blood neutrophils

Neutrophils were isolated from the peripheral blood samples of RA patients and healthy controls by Ficoll-Paque density gradient centrifugation (GE Healthcare, Buckinghamshire, UK) followed by the sedimentation of red blood cells in 6% dextran (Sigma-Aldrich, St. Louis, MO, USA). The residual red blood cells were lysed in a hypotonic solution. The viability of the isolated neutrophils was routinely  > 83%, as confirmed by flow cytometry analysis. The freshly isolated neutrophils were placed into RPMI-1640 culture medium (Thermo Fisher, Waltham, MA, USA) supplemented with 10% heat-inactivated, sterile filtered fetal bovine serum, GlutaMax (0.2%) and HEPES solution (0.2%), (all from Sigma-Aldrich, St. Louis, MO, USA).

### In vitro experiments on peripheral blood neutrophils

To analyse the basal secretion of S100A11, freshly isolated neutrophils from healthy controls (n = 8–10) and RA patients (n = 12–14) were seeded in six-well plates (1 × 10^6^ cells per well) in supplemented RPMI-1640 culture medium (Thermo Fisher, Waltham, MA, USA) and were incubated without treatment for 4 h and 24 h at 37 °C in a 5% CO_2_ atmosphere. Subsequently, the cell culture supernatants were collected and stored at − 80 °C until analysis.

For the stimulation experiments, neutrophils isolated from RA patients (n = 12–14) or healthy controls (n = 8–10) were seeded in six-well plates (5 × 10^5^ per well) and treated with LPS (100 ng/ml, LPS from *E. coli* O26:B6, Sigma-Aldrich, St. Louis, MO, USA) or S100A11 (1000 ng/ml, BioVendor, Brno, Czech Republic) under the culture conditions described above. After 4 h intervals, the supernatants were collected and stored at − 80 °C.

The LPS serotype O26:B6 was selected based on the study by Pieterse et al. that showed species- and serotype-specific differences in the capacity of LPS to trigger NET formation^21^. The dose of recombinant S100A11 was selected in accordance with our previous experiments^16^.

To induce NETosis, neutrophils from RA patients (n = 12–14; 1 × 10^6^ per well) were placed into supplemented RPMI-1640 culture medium (Thermo Fisher, Waltham, MA, USA) and stimulated with PMA (100 nM, Sigma-Aldrich, St. Louis, MO, USA) or DPI (10 μM, Sigma-Aldrich, St. Louis, MO, USA). After 4 h intervals, the supernatants were collected and processed as previously described. To inhibit PMA-induced NETosis, the cells were pretreated with DPI 15 min prior to PMA stimulation.

### Analysis of S100A11, cytokines and NETosis markers

The levels of S100A11 in the synovial fluid or in the supernatants were measured by commercially available ELISA kit (Ray Biotech, Norcross, GA, USA). The detection limit of the assay was 0.03 ng/ml, the interassay and intraassay reliability < 12% and < 10%, respectively. The levels of MPO and citH3 in the synovial fluid or in the supernatants were analysed by commercially available ELISA kits (Abcam, Cambridge, UK and Cayman Chemical, Ann Arbor, MI, USA). The levels of IL-6, TNF and IL-1β in the supernatants were analysed by high sensitivity ELISA kits (Invitrogen, Carlsbad, CA, USA) according to the manufacturer’s instructions. The analysis was performed at 450 nm using a Sunrise ELISA reader (Tecan, Salzburg, Austria). The activity of PADs in the synovial fluid was measured by a home made enzyme-linked immunosorbent assay, where human fibrinogen was used as the immobilized substrate for citrullination and anti-citrullinated fibrinogen antibody as the detecting agent [for more detail see ref.^42^].

### Immunofluorescence double-staining of S100A11 and MPO in the RA synovial tissue

Synovial tissue samples from RA patients (n = 8; 5 females and 3 males, mean age ± SD 67.63 ± 11.35 years) were obtained by biopsies from the hand (n = 3), knee (n = 2), elbow (n = 2) and hip (n = 1) joints. For the double-staining experiments, formalin-fixed and paraffin-embedded synovial sections from RA patients were used and processed as previously described^16^. Then, the samples were incubated with a primary rabbit anti-human antibody against S100A11 (diluted 1:200, Abcam, Cambridge, UK) and a mouse anti-human antibody against MPO (diluted 1:200, Invitrogen, Carlsbad, CA, USA) overnight at 4 °C. The samples were washed with PBS and treated with Alexa 488-conjugated goat anti-rabbit IgG and Alexa 647-conjugated goat anti-mouse IgG secondary antibodies (both diluted 1:200, Abcam, Cambridge, UK) at room temperature (RT, 1 h). The samples were then washed and stained with 4′,6-diamidino-2-phenylindole (DAPI, diluted 1:1000, RT, 10 min, Invitrogen, Carlsbad, CA, USA). Finally, the samples were washed with distilled water and mounted with Fluoromount aqueous mounting medium (Sigma-Aldrich, St. Louis, MO, USA).

### Visualisation of S100A11 in NETs by immunofluorescence

To visualise NETs and S100A11, a suspension of neutrophils (15 × 10^4^ per well) was seeded onto a Nunc Lab-Tek Chamber Slide system (Sigma-Aldrich, St. Louis, MO, USA) and treated with PMA, DPI or LPS as described above in the in vitro stimulation section. After a 4 h incubation interval, the cells were fixed with 4% formalin solution, permeabilized with 1% Triton X (Sigma-Aldrich, St. Louis, MO, USA) and incubated with primary antibodies against MPO and S100A11, secondary antibodies and DAPI, all as described above for the synovial tissue samples. The cells were washed with distilled water and mounted with Fluoromount aqueous mounting medium (Sigma-Aldrich, St. Louis, MO, USA). Images were acquired on a BX53 microscope with a DP80 Colour lens camera, and the images were analysed using cellSens Standard software V1.18 (Olympus, Philadelphia, PA, USA).

### Statistical analysis

The data were statistically analysed using the Wilcoxon test and paired t test for paired data or the Mann–Whitney test for non-paired data. The relationships between non-normally distributed variables were determined by calculating the Spearman correlation coefficient. The data are presented as the median (IQR). *P* values below 0.05 were considered statistically significant (*p < 0.05, **p < 0.01, ***p < 0.001). GraphPad Prism 6 (GraphPad Software, San Diego, CA, USA) was used to perform the analysis.

## Supplementary Information

Supplementary Information

## Data Availability

The datasets used and/or analysed during the current study are available from the corresponding author on reasonable request.

## Abbreviations

Anti-CCP: Anti-cyclic citrullinated peptide antibody
citH3: Citrullinated histone 3
CRP: C-reactive protein
CTRL: Unstimulated controls
DAMPs: Damage associated molecular patterns
DAPI: 4′,6-Diamidin-2-fenylindol
DAS28: Disease activity score in 28 joints from CRP
DMARDs: Disease-modifying antirheumatic drugs
DMSO: Dimethyl sulfoxide
DNA: Deoxyribonucleic acid
DPI: Diphenylene iodonium
ELISA: Enzyme-linked immunosorbent assay
GCs: Glucocorticoids
HC: Healthy controls
IF: Immunofluorescence
Ig: Immunoglobulin
IL: Interleukin
IQR: Interquartile range
LPS: Lipopolysaccharide
MPO: Myeloperoxidase
NADPH: Nicotinamide-adenine dinucleotide phosphate
NETs: Neutrophil extracellular traps
NS: Non-significant
OD: Optic density
PADs: Peptidylarginine deiminases
PBMCs: Peripheral blood mononuclear cells
PBS: Phosphate-buffered saline
PMA: Phorbol 12-myristate 13-acetate
RA: Rheumatoid arthritis
RAGE: Receptor for advanced glycation end products
RF: Rheumatoid factor IgM
RPMI: Roswell Park Memorial Institute
RT: Room temperature
SEM: Standard error of the mean
SD: Standard deviation
TLR: Toll-like receptor
TNF: Tumour necrosis factor
