# Supplementary material for: S100A11 (calgizzarin) is released via NETosis in rheumatoid arthritis (RA) and stimulates IL-6 and TNF secretion by neutrophils
Source: Sci Rep. 2021 Mar 16;11:6063. doi: 10.1038/s41598-021-85561-3 (PMC7966750; doi:10.1038/s41598-021-85561-3)
Supplement: Supplementary file 1 — Supplementary Information [file 41598_2021_85561_MOESM1_ESM.pdf]

S100A11 (calgizzarin) is released via NETosis in rheumatoid arthritis (RA) and stimulates IL-6 and TNF secretion by neutrophils

Adéla Navrátilová[1,2], Viktor Bečvář[1], Jiří Baloun[1], Dres Damgaard[4], Claus Henrik Nielsen[4], David Veigl[3], Karel Pavelka[1,2], Jiří Vencovský[1,2], Ladislav Šenolt[1,2], *Lucie Andrés Cerezo[1,2]

[1]Institute of Rheumatology, Prague, Czech Republic

[2]Department of Rheumatology, 1st Faculty of Medicine, Charles University, Prague, Czech Republic

[3]First Orthopaedic Clinic, 1st Faculty of Medicine, Charles University, Prague, Czech Republic

[4]Institute for Inflammation Research, Center for Rheumatology and Spine Diseases, Copenhagen University Hospital, Rigshospitalet, Copenhagen, Denmark

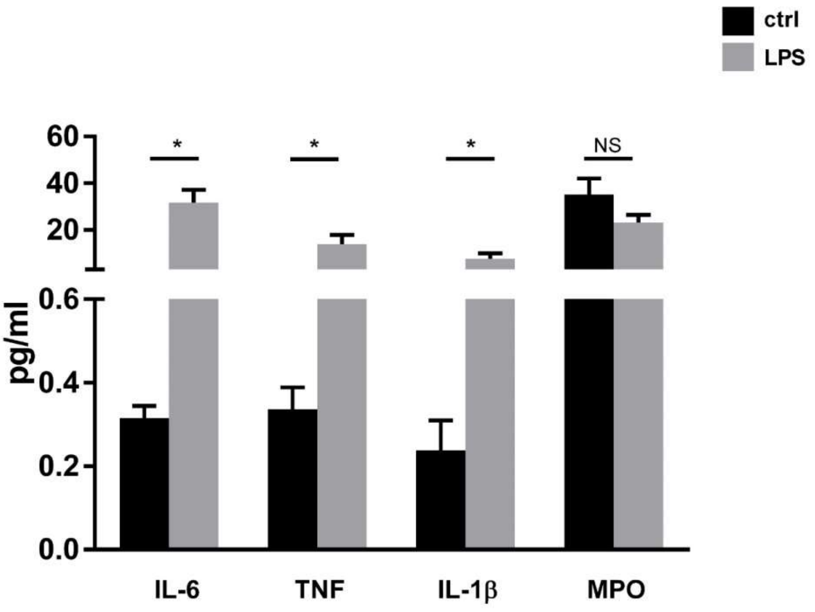

Supplementary figure S1 Lipopolysaccharide (LPS) from E.coli serotype O26:B6 used in our *in vitro* experiments stimulates inflammatory response in RA neutrophils by inducing secretion of IL-6, TNF and IL-1β, but not NETosis (assessed by the levels of MPO), (n=7). Ctrl, unstimulated control; NS, non-significant. Data are represented as mean±SEM. *$p<0.05$
